# A Compact Annular Ring Microstrip Antenna for WSN Applications

**DOI:** 10.3390/s120708663

**Published:** 2012-06-26

**Authors:** Daihua Wang, Linli Song, Hanchang Zhou, Zhijie Zhang

**Affiliations:** 1 National Key Laboratory for Electronic Measurement Technology, North University of China, Taiyuan 030051, China; E-Mail: zhangzhijie@nuc.edu.cn; 2 Key Laboratory of Instrumentation Science and Dynamic Measurement, Ministry of Education, North University of China, Taiyuan 030051, China; E-Mails: solili@163.com (L.S.); zhc04@yahoo.com.cn (H.Z.)

**Keywords:** microstrip antenna, resistor loading, effects of metal on antenna characteristics

## Abstract

A compact annular ring microstrip antenna was proposed for a wireless sensor network (WSN) application in the 2.4 GHz band. In this paper the major considerations of the conformal antenna design were the compact size and the impact on antenna's performance of a steel installation base. By using a chip resistor of large resistance (120 Ω) the antenna size was reduced to 38% of that a conventional annular ring patch antenna. With the addition of the steel installation base the resonant frequency of the antenna increases about 4.2% and the bandwidth reduces from 17.5% to 11.7% by adjusting the load resistance simultaneously. Several key parameters were discussed and optimized, and the antenna was fabricated and its performance measured. The antenna is well matched at 2.4 GHz with 34.2 dB return loss and –2.5 dBi peak gain. Meanwhile, it exhibits excellent radiation patterns with very low cross-polarization levels.

## 1. Introduction

Microstrip patch antennas have been widely used in many systems [1–4] because of their attractive features such as compact size, low profile, light weight and ease of fabrication [5–9]. Various shapes of the patches were proposed for these antennas, such as circular, square, triangular, elliptical, diamond, *etc.* [6–8,10,11]. However, in some applications the small size conventional patch antenna is still too large [5,12–14], so research is still being focused on the miniaturization of the patch antenna over the years. At present, there were many effective size reduction methods, including using shorting pins, resistor load, high dielectric constant materials and slots, *etc.* [5–8,15–18].

**Figure 1.**
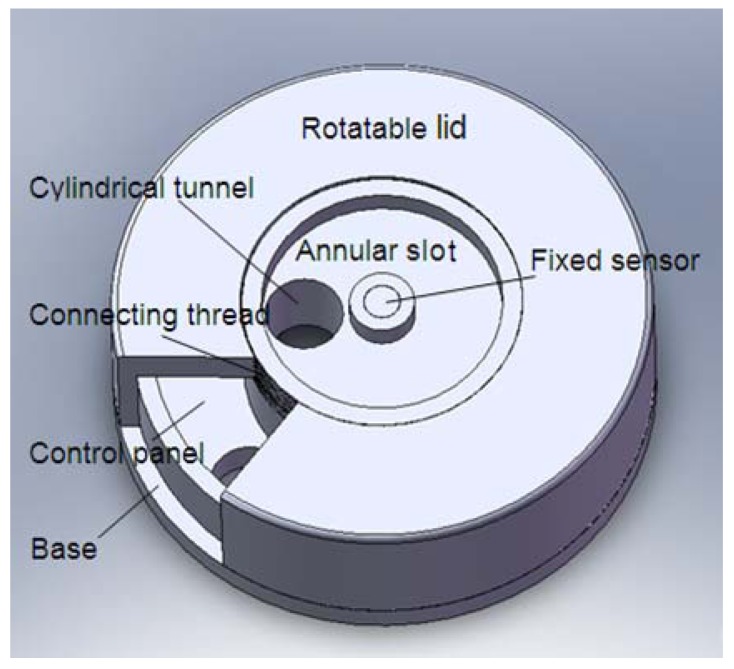
Installation base of the antenna.

In this paper, conformal antenna design with compact size was one major consideration. The antenna was proposed for a wireless sensor network (WSN) application in the 2.4 GHz band. The sensor nodes executed monitoring in an explosive experiment for military use. The structure for the antenna installation was designed as a part shell of the sensor node which is illustrated in Figure 1. The materials of all components were steel to resist physical damage in the explosive experiment. The wireless transceiver module was installed beneath the installation base of the antenna and protected by a surrounding steel shell. To propagate out RF signals from the cage the only approach was a coaxial cable through a cylindrical tunnel on the installation base. The patch antenna could be installed only in a thin annular slot on the top of the installation base whose inner radius was greater than 5 mm and outer radius less than 20 mm. Therefore, the size of the designed antenna must comply with the requirements of the limited mounting space.

Another main consideration of the research was the impact on the antenna's performance of the steel installation base. In recent years the influences of metal carriers on conformal antenna design were studied [19,20], however, there were less concerns about planar antenna design. For most reported microstrip antennas the surroundings were usually considered free space. In our studies the steel installation base greatly influenced the characteristics of the proposed antenna such as resonant frequency, bandwidth and radiation characteristics, *etc.*, so how to design the metal carrier was an important issue in the antenna design.

The configuration of the proposed antenna and the parameter settings are described in Section 2. To meet the compact and conformal requirements an annular ring patch antenna with a loaded chip resistor was designed. In Section 3, key parameters were studied, especially for the outer radius of the substrate and the patch, position of the loading point, and the resistance of the chip resistor. Then the proposed antenna was manufactured and the measured results were compared with the simulation ones. Finally, the conclusions are summarized in Section 4.

## 2. Antenna Configuration and Design

**Figure 2.**
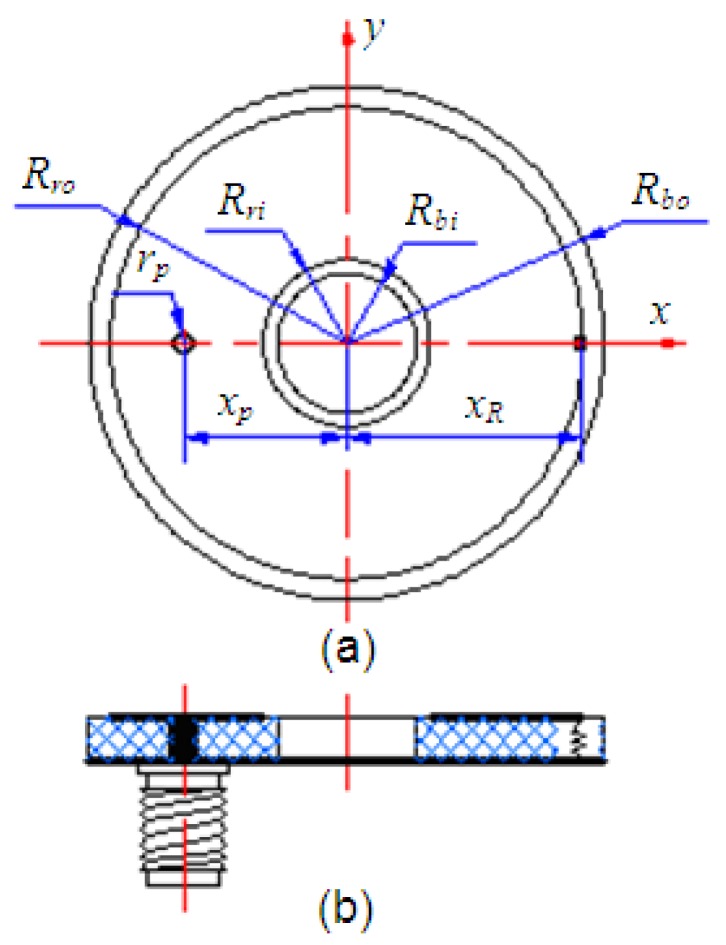
Configuration and parameters of the proposed antenna. (**a**) Top view. (**b**) Cross section.

The schematic of the antenna configuration is shown in Figure 2. It was designed as an annular ring structure with outer radius of *R_bo_* (≤20 mm), inner radius of *R_bi_* (≥5 mm), and thickness of *h*. To reduce costs FR4 substrate with relative dielectric constant of *ε_r_* = 4.5 and loss tangent of tanδ = 0.02 was selected. On the top side of the substrate, an annular ring radiating patch with outer radius of *R_ro_* inner radius of *R_ri_* and metallization thickness of 35 μm was printed. The ground plane was placed full size on the opposite side of the substrate. As shown in Figure 2 the antenna was back fed by a coaxial probe of radius *r_p_* located at a distance of *x_p_* away from the circular center. The cylindrical tunnel in the installation base shown in Figure 1 was designed as the access for RF signal propagation through the coaxial probe. The diameter of the cylindrical tunnel was just suit for the coaxial probe, while almost as large as the width of the annular slot due to the compact space. So the distance of *x_p_* was fixed around the center of the annular slot. It was shown in [14,21] that for minimum patch area the feed point must be located nearest to the radiating edges of the patch. The criterion in our project cannot be achieved, which brings more burden to the miniaturization.

As a conventional annular ring patch antenna with parameter settings above mentioned, the outer radius of *R_bo_* is at least 30 mm at resonant frequency of 2.4 GHz with very narrow bandwidth (as low as 1.7%). It is obvious that a conventional annular ring patch antenna is not suitable for the limited mounting space. As mentioned in Section 1, loading is an effective way to reduce antenna size. In this design, a chip resistor of resistance *R* was loaded along the x-axis with a distance of *x_R_* away from the circular center and the parameters *R* and *x_R_* were selected carefully for impedance matching.

The thickness of the substrate (*h*) was another key parameter for design. Increasing the thickness the bandwidth and the efficiency of the patch antenna was improved [22], while the unwanted surface waves were further excited too [14]. In view of the limited mounting space and the factors above mentioned, the thickness of the proposed antenna was fixed at *h* = 3 mm.

## 3. Results and Discussion

To optimize antenna's characteristics, such as resonant frequency, bandwidth, gain, radiation efficiency and level of cross-polarization, a series of parameters of the annular ring antenna should be carefully selected. In this section, the effects of several key parameters on the proposed antenna were discussed and simulated using the Ansoft HFSS. In comparison with the simulated results, several antennas were fabricated and measured.

### 3.1. Effects of Key Parameters in Free Space

#### 3.1.1. Effect of the Antenna Dimensions

**Figure 3.**
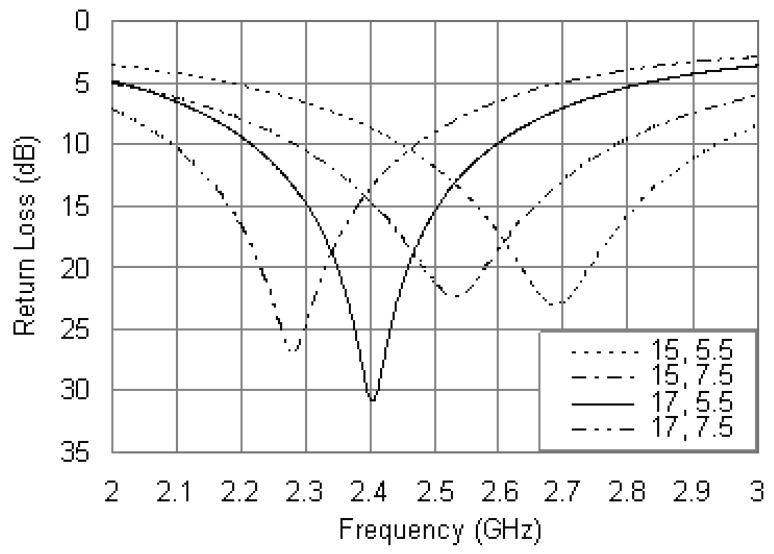
Simulated return loss curves with different values of *R_ro_* and *R_ri_*.

Figure 3 shows the simulated return loss plots for different dimensions of the annular ring with fixed parameters as *R_bi_* = 5 mm, *R_bo_* = 18.5 mm, *r_P_* = 0.6 mm, *x_p_* = –12 mm, *x_R_* = *R_ro_* – 0.5 mm, and *R* = 56 Ω. From the simulated results of Figure 3, it is found that the resonant frequency is very sensitive to the patch size. As the outer radius (*R_ro_*) or inner radius (*R_ri_*) increases, the resonant frequency is decreased. The antenna impedance is well matched at 2.4 GHz with the combination of *R_ri_* = 5.5 mm and *R_ro_* = 17 mm, and the impedance matching is degraded while the patch dimensions deviate from the optimum values. Compared with the conventional annular ring patch antenna above mentioned, Figure 3 shows that a greater bandwidth can be obtained. There is also a notable decrease in the bandwidth as the outer radius (*R_ro_*) increases, while the inner radius (*R_ri_*) causes negligible effects.

In the process of analysis, it is also found that in comparison with the patch size the substrate size has relatively less effects on the resonant frequency. The resonant frequency slightly decreases with increasing outer radius (*R_bo_*). To meet the compact requirements of the antenna, the outer radius of the substrate (*R_bo_*) was selected as 18.5 mm with consideration of the optimized performance. The inner radius of the substrate (*R_bi_*) was fixed at 5 mm due to the installation requirement of the sensor (shown in Figure 1).

#### 3.1.2. Effect of the Loading Position

**Figure 4.**
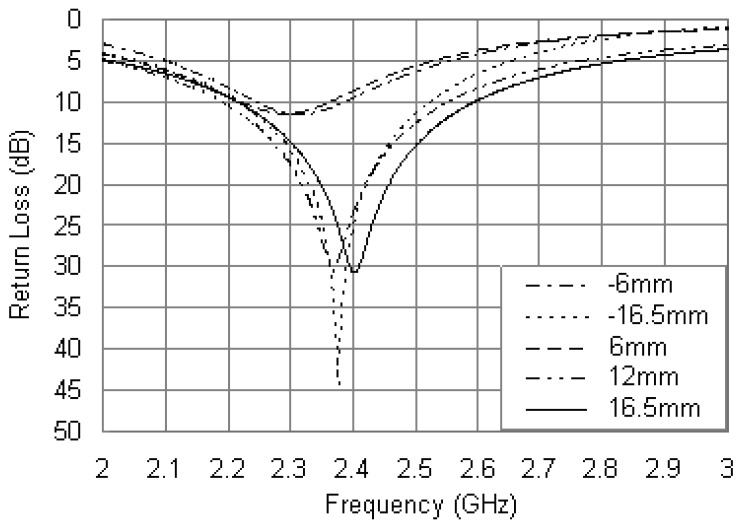
Simulated return loss curves with different values of *x_R_*.

To minimize the dimensions of the proposed antenna and increase the operating bandwidth, a chip resistor was introduced into the design to adjust the input impedance characteristics. When modeling in HFSS, a thin two-dimensional surface (0.3 mm × 3 mm) was placed in parallel with the z-axis at the loading position between the patch and the ground, and a lumped RLC boundary was assigned in parallel on the surface. Figure 4 shows the return loss curves for different positions of the loading point with other parameters of *R_ri_* = 5.5 mm, *R_ro_* = 17 mm, *r_P_* = 0.6 mm, *x_p_* = –12 mm and *R* = 56 Ω. There is an upward shift for the resonant frequency as the loading point is moved away from the circular center with the bandwidth improved significantly, and a notable agreement between the loading points at same radius along the x-axis is also observed. It was shown in [14,21] that for minimum area the loading point should be located closest to one of the radiating edges of the patch. As seen from Figure 4, the case of loading at the inner edge of the patch can result in a further reduction in resonant frequency, which corresponds to an even larger size reduction at a given operating frequency. In this case, the resistor loading has maximum effects on the resonant-frequency lowering due to the surface current has a maximum value around the inner edge of the patch, however, the matching level is poor. With consideration of the symmetrical characteristics [23] and easy of fabrication, the loading point was placed at the outer edge of the patch with *x_R_* = 16.5 mm.

#### 3.1.3. Effect of the Loading Resistance

**Figure 5.**
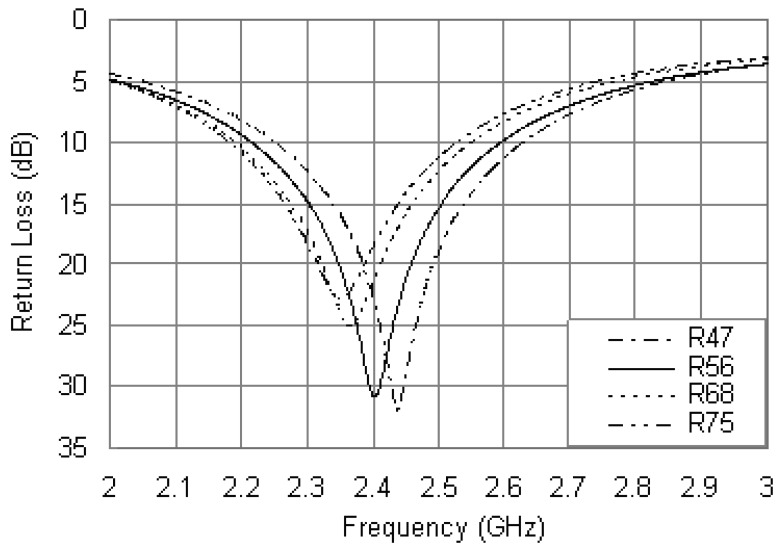
Simulated return loss curves with different values of *R*.

Another important parameter is the resistance of the loading resistor. Efforts were made to optimize the value of *R* to obtain optimum impedance matching for the proposed antenna. Figure 5 shows the return loss curves for different values of *R* and fixing other parameters as: *R_ri_* = 5.5 mm, *R_ro_* = 17 mm, *r_P_* = 0.6 mm, *x_p_* = –12 mm and *x_R_* = 16.5 mm. It is evident that the resonant frequency of the antenna is dependent on the resistance of the loading resistor. As the *R* value increases, the center frequency decreases with the matching level slightly affected. No significant change in bandwidth is observed with the changes of *R*. Hence, the value of *R* can be adjusted to provide better resonant mode and good matching.

In comparison with the previously reported designs [24–26], an important change of this design, which kept the antenna operating at required frequency while allowing dramatically reduced dimensions, was the large resistance of the loading resistor. Several resistors with small resistance less than 10 Ω were analyzed, and the resonant frequencies were higher than that one in Figure 5 with poor matching levels. For example, the center frequency is 2.65 GHz as the resistance of *R* = 1 Ω, and the return loss is as low as 4.7 dB. In this case, the matching conditions are destroyed, and the impedance is mismatched. However, it should be noted that to reduce antenna dimensions the introducing resistor of large resistance was at the expense of the radiation efficiency, similar to the observation in [25–27]. The radiation efficiency of the conventional annular ring antenna above mentioned is 24.9%, with the introducing resistor (*R* = 56 Ω), the radiation efficiency of the proposed antenna is reduced to 14.6% and the peak gain is –1.5 dBi.

### 3.2. Measurement and Comparison

**Figure 6.**
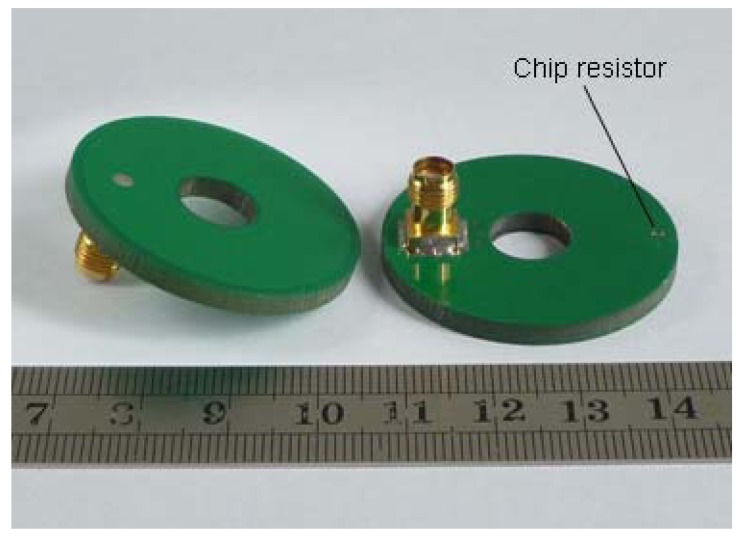
Photograph of the fabricated antenna.

**Figure 7.**
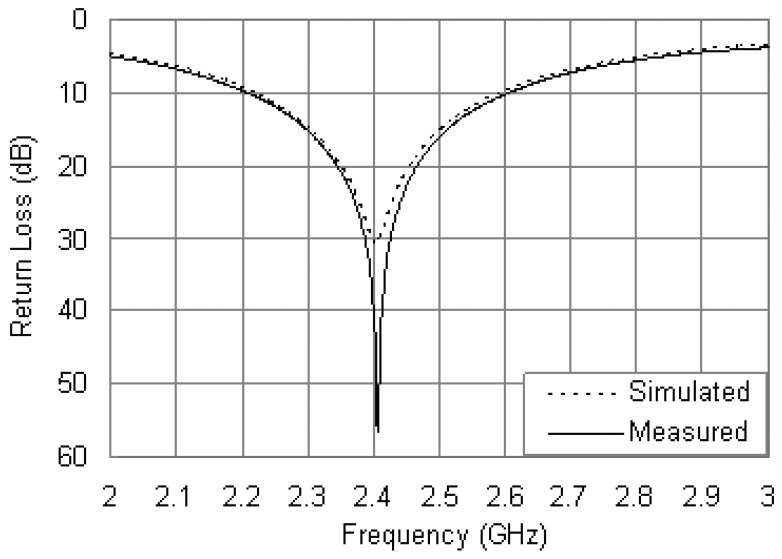
Measured and simulated return loss curves.

The fabricated prototype of the proposed antenna is shown in Figure 6. A chip resistor in 0402 package was mounted directly onto the bottom surface of the PCB. The loading terminal of the chip resistor was connected to the radiating patch through a small via hole, and the opposite side was directly connected to the ground plane. The via hole was drilled at the position of *x_R_* = 16.5 mm and with a diameter was 0.3 mm which complied with the simulation settings. The only variation between the fabricated prototype and the simulation model was the mounting method of the resistor, and the following results showed that the influence of the variation was negligible. The measured and simulated return loss curves are compared in Figure 7. Both the measured and simulated curves are excited at 2.4 GHz with return loss as 56.7 and 30.8 dB, respectively. The measured impedance bandwidth is 17.5% from 2.20 to 2.62 GHz, and the simulated impedance bandwidth is 16.3% from 2.21 to 2.60 GHz. The measured results show good agreement with the simulated ones in Figure 7.

**Figure 8.**
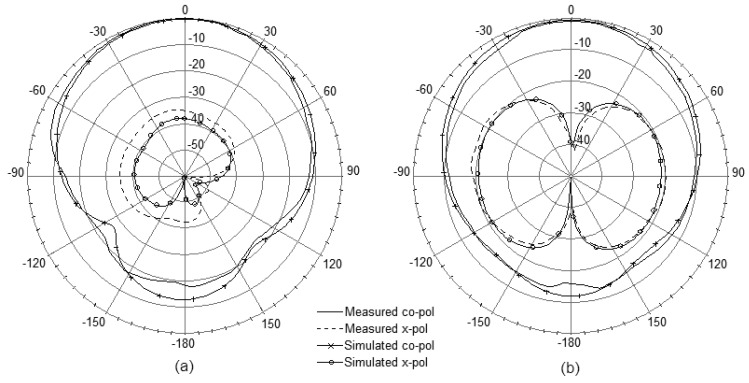
Measured and simulated radiation patterns. (**a**) E-plane. (**b**) H-plane.

Figure 8 presents the measured and simulated radiation patterns including the co-polarization and cross-polarization in the E- and H-plane. From the measured results the radiation patterns are symmetric and there is minimal ripple in both E- and H-plane which are in good agreement with the simulation. The antenna also shows good cross-polarization level as low as –20 dB. However, due to a small ground-plane size, backward radiations are observed obviously in both planes.

### 3.3. Effects of the Steel Installation Base

Due to the limited mounting space, the characteristics of the proposed antenna were influenced inevitably. In this subsection, the steel installation base was introduced into the simulation model of the antenna in Ansoft HFSS.

#### 3.3.1. Effect on Impedance Characteristics

**Figure 9.**
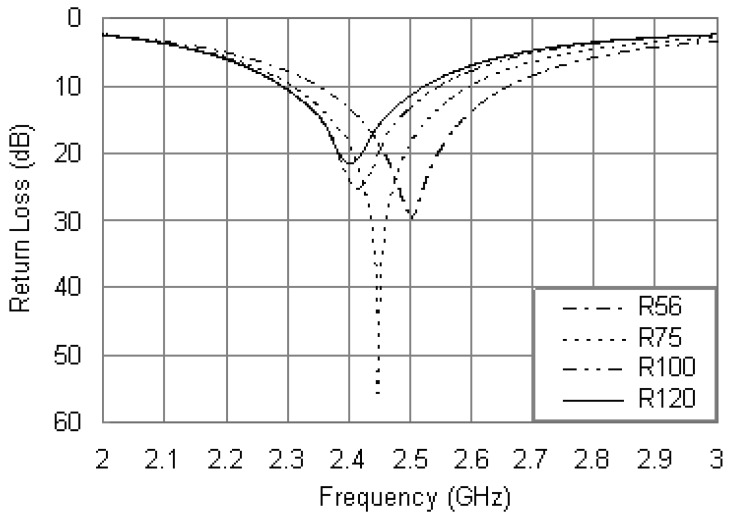
Simulated return loss curves of the proposed antenna along with the installation base.

Figure 9 displays the simulated return loss curves of the proposed antenna with the steel installation base, and other parameters were set as the same values in Figure 5. By comparing the simulated results in Figure 9 and Figure 5, it is observed that when the steel installation base was introduced, the resonant frequencies were obviously increased. Taking the case of *R* = 56 Ω as an example, the resonant frequency in Figure 5 is 2.40 GHz with a return loss of 30.8 dB, while in Figure 9 that is 2.50 GHz with a return loss of 29.6 dB. Consequently, the addition of the steel installation base further strengthens the inductive nature of the patch antenna and hence increases the operating frequency, which makes it more difficult to realize the required compactness. Another variation between the results of Figure 9 and Figure 5 is the reduced bandwidth. As above mentioned, the simulated bandwidth is 16.3% in the case of *R* = 56 Ω, while it is reduced to 13.6% from 2.34 to 2.68 GHz due to the influence of the steel installation base.

In order to obtain a good impedance matching for the 2.4 GHz, as can be seen in Figure 9, the resistance of the loading resistor can be improved. It is observed that for *R* = 120 Ω, the antenna with the steel installation base resonates at 2.4 GHz with a return loss of 22.1 dB, and the bandwidth is 10.4% from 2.28 to 2.53 GHz.

**Figure 10.**
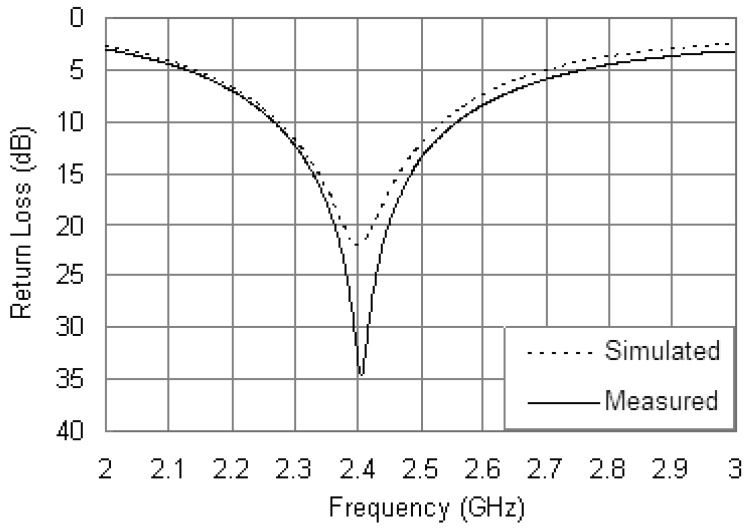
Measured and simulated return loss curves of the antenna along with the installation base.

Figure 10 shows the measured and simulated return loss curves of the antenna with the steel installation base for comparison. It is found that the measured resonant frequency is 2.4 GHz with a return loss of 34.2 dB, and the measured bandwidth is 11.7% from 2.27 to 2.55 GHz. Good agreement is achieved between the measured and simulated results. The slight discrepancy in the measured and simulated curves can be attributed to the fabrication tolerances.

#### 3.3.2. Effect on Radiation Characteristics

**Figure 11.**
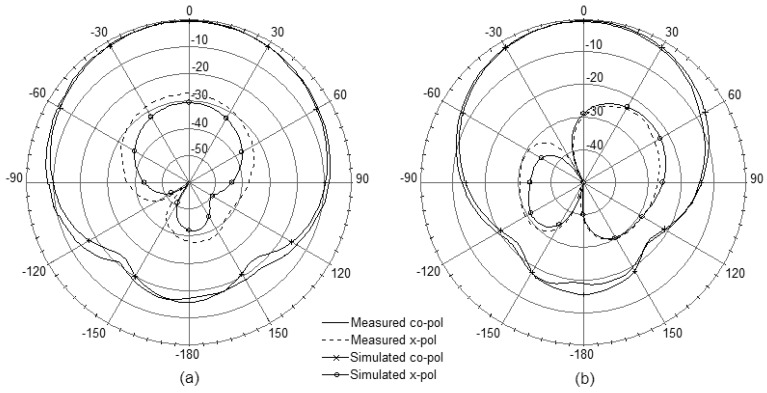
Measured and simulated radiation patterns of the antenna along with the installation base. (**a**) E-plane. (**b**) H-plane.

The far-field patterns in both the E- and H-plane were simulated and measured, and the results are shown in Figure 11. In comparison with the results shown in Figure 8, the symmetric characteristics in co-polarization patterns are maintained with the polarization levels slightly reduced. Simultaneously, the backward radiations are weakened in both the planes due to the backed steel installation base, which can be explained as the restraining effects of the metal material. It is also noted that the cross-polarization patterns are changed obviously between Figure 11 and Figure 8, while the polarization levels are remained well.

**Figure 12.**
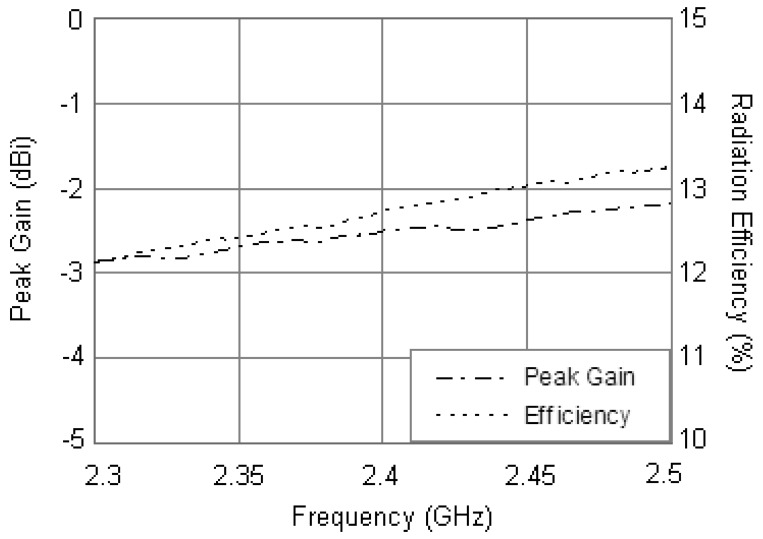
Measured peak gain and radiation efficiency of the antenna along with the installation base.

The measured peak gain of the antenna backed with the installation base is plotted in Figure 12 along with its radiation efficiency. The peak gain is between –2.8 and –2.2 dBi within the operating band (2.3–2.5 GHz) with the radiation efficiency is from 12.2% to 13.3%. For the center frequency of 2.4 GHz, with the influences of installation base and resistor loading, the radiation efficiency of the antenna reduces to 12.7% and the peak gain is –2.5 dBi.

For further study to improve the gain of the antenna a substrate with relatively low permittivity can be used and its thickness increased suitably, however, the gain improvement may be relatively limited. Another approach to obtain an enhanced antenna gain is by covering the FR4 substrate with a high permittivity superstrate layer, and its thickness needs to be optimized to gain of the required compactness of the antenna. Furthermore, it may be possible to increase the gain by incorporating suitably meandered geometries.

## 4. Conclusions

In this paper, a compact annular ring mirostrip antenna backed with a steel installation base was realized. By using a loading resistor of large resistance, 38% miniaturization is achieved compared with a conventional annular ring antenna. It is also found that the steel installation base has a great influence on the antenna's characteristics. With the addition of the steel installation base, the resonant frequency of the antenna increases about 4.2%, while the measured bandwidth was reduced from 17.5% to 11.7% by simultaneously optimizing the load resistance. The realized antenna is well matched at 2.4 GHz with good radiation characteristics. It should also be noted that, due to the dramatic size reduction and influences of the steel installation base, the radiation efficiency reduces from 24.9% to 12.7% and the peak gain is –2.5 dBi. The configuration of proposed antenna is simple and easy to fabricate. Several antennas have been used in the WSN successfully and the performance has been verified. The design provides a reasonable solution to the applicability of microstrip antenna used in narrow spaces.
